# A Finger Grip Force Sensor with an Open-Pad Structure for Glove-Type Assistive Devices

**DOI:** 10.3390/s20010004

**Published:** 2019-12-18

**Authors:** Junghoon Park, Pilwon Heo, Jung Kim, Youngjin Na

**Affiliations:** 1 Department of Mechanical Engineering, Korea Advanced Institute of Science and Technology (KAIST), Daejeon 34141, Korea; junghoon.park@kaist.ac.kr (J.P.); pwheo@kaist.ac.kr (P.H.); jungkim@kaist.ac.kr (J.K.); 2 Department of Mechanical Systems Engineering, Sookmyung Women’s University, Seoul 04310, Korea

**Keywords:** fingertip force, open-pad structure, capacitive sensor

## Abstract

This paper presents a fingertip grip force sensor based on custom capacitive sensors for glove-type assistive devices with an open-pad structure. The design of the sensor allows using human tactile sensations during grasping and manipulating an object. The proposed sensor can be attached on both sides of the fingertip and measure the force caused by the expansion of the fingertip tissue when a grasping force is applied to the fingertip. The number of measurable degrees of freedom (DoFs) are the two DoFs (flexion and adduction) for the thumb and one DoF (flexion) for the index and middle fingers. The proposed sensor allows the combination with a glove-type assistive device to measure the fingertip force. Calibration was performed for each finger joint angle because the variations in the expansion of the fingertip tissue depend on the joint angles. The root mean square error (RMSE) for fingertip force estimation ranged from 3.75% to 9.71% after calibration, regardless of the finger joint angles or finger posture.

## 1. Introduction

Fingertip forces play an important role in a wide range of activities in daily living, and grip strength (the ability to exert a fingertip force) is closely connected to health-related quality of life [1]. Loss of grip strength can occur for several reasons, including stroke [2], carpal tunnel release surgery [3], aging [4], brachial plexus injury [5], spinal cord injury [6], and work-related musculoskeletal disorders (WMSDs) [7].

To assist reduced grip strength or prevent WMSDs, many assistive hand exoskeletons have been developed [8,9,10,11,12,13,14,15,16] that detect the intention of the wearer’s intention via fingerpad contact forces [17,18,19,20], finger motion [20,21,22,23,24], surface electromyography (sEMG) [25,26,27], or multimodal sensing [28]. Although the measurement of fingerpad contact force enables the acquisition of individual finger forces with simple sensors, the tactile sensation of the wearer is inevitably diminished because of the presence of the force sensor between the fingerpad and object being manipulated. This tactile obstruction may cause excessive grip force or slipping, which can lead to object damage or even injury [29,30] because of the importance of tactile sensation during manipulation dexterity [31,32,33]. Motion-based intention extraction methods necessitate finger movements prior to actuation of the hand exoskeleton; therefore, this approach is not suitable for isometric force exertion. Intention extraction methods using sEMG signals are capable of estimating the grip force of a wearer from the electric potentials present in the related muscles. This approach enables direct contact between the fingerpads and an object. However, there are limitations, such as the high sensitivity of the sEMG signals to skin conditions and electrode locations, motion artifacts, and the requirement for many sensors to have multiple degrees of freedom (DoF), which make this approach impractical [34,35,36]. Although not applied to assistive hand exoskeletons, there are many approaches to obtain the fingertip force using fingernail color [37,38,39] and the deformation of tissue [40,41,42]. Fingertip-mounted devices have also been used to estimate finger movements in interaction devices [43,44].

We previously proposed a grip force sensing method based on the lateral expansion force that is produced by exerting a grip force for an assistive hand exoskeleton [45]. This exoskeleton has one active DoF for the index finger, and the entire exoskeleton was built with rigid materials. With tactile sensation, the fingertip force magnitude could be adjusted during grasping tasks and successfully maintained a static force. However, this previously developed sensor has a few limitations. First, this sensor design was developed only for the index finger, not including a thumb. Second, commercially available load cells were used to measure the fingertip force. The design and size of the sensing module ware determined by the specifications of the load cells. Custom sensors must fit on each finger via optimal designs that are required for different applications.

In this study, we developed the fingertip force sensors based on a capacitive sensor, which can be mounted on the distal finger segments using a glove-type assistive device and open fingerpads. A flexible glove-type structure was chosen because it is simple and compact to use. There are no fixed mechanical joints in the developed device because the glove does not have a rigid frame and precise alignment of the joint axes between the hand of the wearer and the device is not required. For these reasons, glove-type hand exoskeletons have been used in several studies [14,15,16,17,18]. The major improvements relative to previous studies are as follows: The number of measurable DoFs was increased to four, flexion of the index and middle fingers and flexion and adduction of the thumb. The proposed fingertip force sensors with embedded electric parts are mounted at the distal segment of each finger for grip force estimation, unlike in our previous system that used load cells.

## 2. Materials and Methods

**Figure 1:**
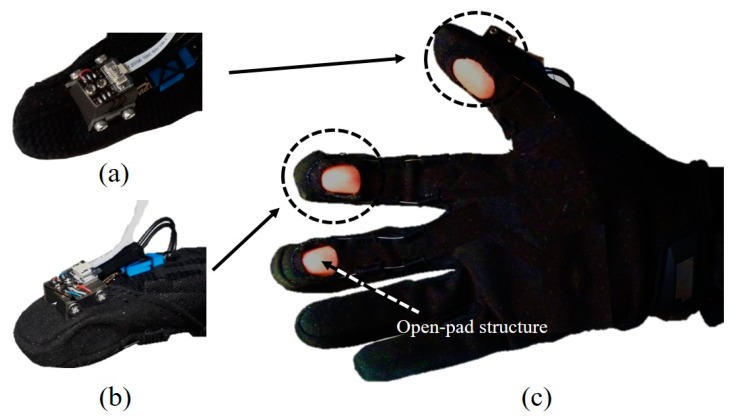
Glove with an indirect fingertip force sensor and an open-pad structure: (**a**) the top-front-right view of the thumb, (**b**) the top-front-right view of the index and middle fingers, and (**c**) the bottom view of the glove with an open-pad structure.

The design of the proposed fingertip force sensor and the evaluation of the sensor performance are described in this section. As shown in Figure 1a,b, the proposed sensors were installed in the fingertips of a glove. The sensors were installed on three fingers (the index, middle, and thumb). From the perspective of grip force assistance, the index and middle fingers and thumb mainly contribute to grasping during multi-fingered grip force exertion [46], especially when a large force is required [47].

For each finger, the proposed sensor is placed on the dorsal side of the distal segment, where two capacitive sensors are attached to the lateral surfaces of the finger. Although the sensor is placed on the dorsal side, we found that the installation of the fingertip sensors on the human finger did not affect a person’s ability to catch objects from our previous research [45]. There are openings on the palmar surfaces of the fingers of the glove so that the fingers can make contact with the object being manipulated, as shown in Figure 1c. This open-pad structure exposes the skin of the wearer at the distal phalanx. From that previous study, the effect of tactile sensation was evaluated based on variation of grip force. With tactile sensation, users can grasp an object with reduced grip force compared to without sensation. The user can detect the sense of touch through the exposed skin, specifically the information received from varying pressure or vibration against the skin from the mechanoreceptors, which can measure high-frequency stimuli up to 300 Hz. If there is the obstacle between the human skin and the object, it is reduced that the frequency range of vibration stimulus human can feel [45]. We selected the size of the hole to be as large as possible so that the fingertip force sensors are completely covered by the glove to protect the developed sensor from the external environment, such as from water and foreign substances. The size of the sensor was based on a subject (male, 26 years, and right-handed), who participated in the grip force measurement experiment in Section 3. His fingertip width and height are 18.5 and 12.3 mm for the thumb, 14.9 and 10.5 mm for the index finger, and 15.8 and 11.9 mm for the middle finger.

### 2.1. Design of the Fingertip Force Sensor

**Figure 2:**
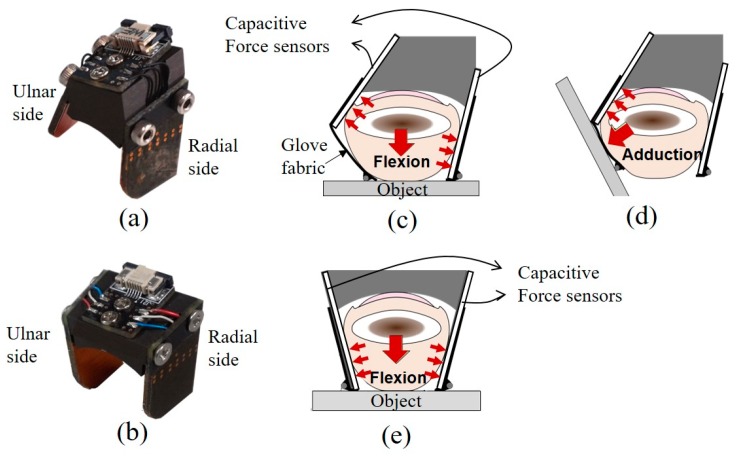
Photographs of the actual fingertip force sensors for (**a**) the thumb; and (**b**) index and middle fingers. Schematic designs for (**c**) thumb flexion sensing, (**d**) thumb adduction sensing, and (**e**) index/middle finger flexion sensing.

The fingertip force sensor for the thumb has a different design from that of the sensors for the index and middle fingers. Unlike other finger joints, the thumb has a carpometacarpal joint or trapeziometacarpal joint whose function is to optimize the hand pinch motion. This joint allows flexion, extension, abduction, and adduction motions; therefore, the fingertip force for the thumb must be measured in both the adduction and flexion DoFs. Figure 2a shows a photograph of the actual thumb sensor. The capacitive sensor on the ulnar side is placed in a more dorsal position than the radial-side capacitive sensor and is inclined outward, whereas the ulnar- and radial-side sensors for the index and middle fingers are placed symmetrically and are inclined inward. The position and orientation of the ulnar-side sensor were determined empirically to enable a comfortable grip posture during adduction and flexion of the thumb. As is the case for the index and middle fingers, the application of a compressive force to the thumb tissue by the phalanx (bone) and the manipulated object in the volar-dorsal direction causes the tissue to expand laterally, as shown in Figure 2c. The tissue expands toward the radial-side sensor and the glove fabric, which is wrapped around the ulnar side of the thumb. The direction of the adduction force, as shown in Figure 2d, is not perpendicular to the direction of the flexion force. Because of the inward incline of the ulnar-side sensor and the friction between the inner surface of the glove and the skin of the wearer, the pressure on the ulnar-side sensor is greater than that on the radial-side sensor. Upon exertion of an adduction force, the phalanx tends to be displaced toward the ulnar side, and compression of the thumb tissue between the object and the phalanx causes the fingerpad tissue to protrude through the palmar opening. Simultaneously, the tissue between the phalanx and the radial-side sensor experiences less pressure.

The fingertip force sensor for the index and middle fingers is shown in Figure 2b. The large red arrow in the center of Figure 2e indicates the flexion force exerted by the wearer. The application of a compressive force on the finger tissue by the phalanx and the object in the volar-dorsal direction causes lateral expansion of the tissue, and pressure is exerted on the capacitive sensors, which resists lateral expansion of the finger tissue. The proposed sensors were constructed from hard-anodized aluminum, and the anodization provides electrical insulation to prevent electrical shorting of the capacitive sensors.

In the case of the thumb, the shear force generated when gripping a heavy object can be measured on the ulnar side of the fingertip force sensor. As shown in Figure 2d, the object is held by the force along the direction of thumb adduction. However, we did not measure the force in the shear direction for the index and middle fingers because for these fingers, our aim was to measure the grip force in only one DoF (flexion/extension). Previously, we analyzed the effect of the shear forces that arise when gripping an object using our hard hand exoskeleton [28]. It is possible to analyze the effect of the shear force on the sensor in the case of this hard hand exoskeleton in which fingertip force sensors with load cells are installed in a rigid linkage that covers the entire hand and is firmly fixed to the hand. However, it is more difficult to investigate the effect of the shear force for a soft hand exoskeleton. Since the fingertip force sensor is installed in a soft glove, the sensor may rotate, meaning that the force cannot always be measured at the same level. 

### 2.2. Capacitive Sensors

**Figure 3:**
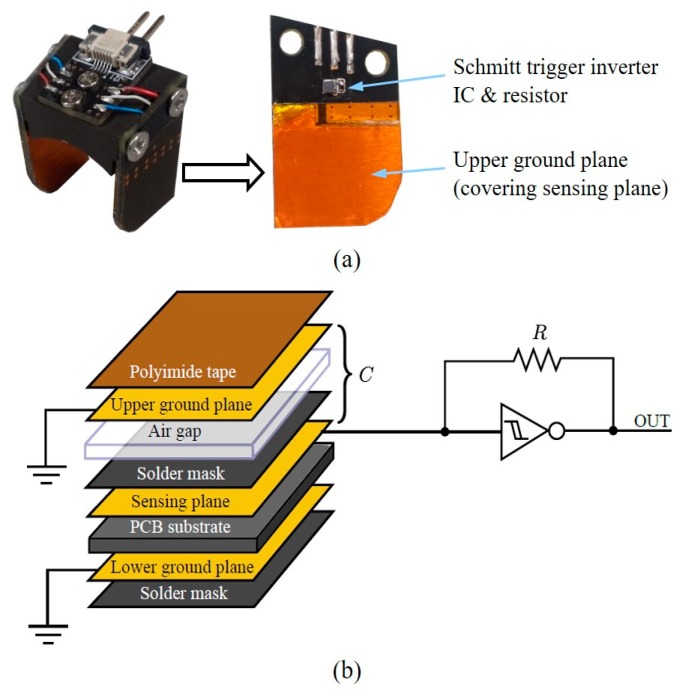
Fingertip force sensor configuration with the capacitive sensors. (**a**) The fabricated capacitive sensor mounted on the fingertip force sensor. (**b**) The capacitive sensor configuration with a multi-vibrator circuit using a Schmitt trigger inverter to measure the oscillation period.

Capacitive sensors were selected to measure the fingertip force because of their high sensitivity, high dynamic range, and low hysteresis [48,49]. The mechanism of capacitive force sensing depends on two parallel plates separated by a distance that changes as a normal force is applied. The capacitance is expressed as follows:

(1) $$C=\varepsilon \frac{A}{d},$$

where *C*, *ε*, *A*, and *d* are the capacitance between the parallel plates, the permittivity, the area of the plates, and the distance between the plates, respectively. A change in *d* induced by an external force causes a change in *C*, which can be measured to estimate the applied force because the permittivity and area of the plates are constant. Capacitive sensors, which are composed simply of a ground plate and a sensing plate, are susceptible to environmental noise and can be affected by stray capacitances between the sensing plate and other objects. Therefore, a multilayer configuration was chosen, as presented in Figure 3a [50]. A multi-vibrator circuit based on a Schmitt trigger inverter was used to convert the change in capacitance into a frequency shift. In this multi-vibrator circuit, as shown in Figure 3b, the capacitor repeatedly charges and discharges at a specific frequency that is determined by the capacitance, *C*, and the resistance, *R*, as follows:

(2) $$f=\frac{1}{T}\approx \frac{1}{K\cdot R\cdot C},$$

where *f*, *T*, and *K* are the sensor output oscillation frequency, the sensor output oscillation period, and a factor that depends on the characteristics of the specific inverter IC and its operating voltage, respectively. Under the assumption that *A* and *ε* in Equation (1) and *K* and *R* in Equation (2) are constant, *f* depends only on *d* (the distance between the plates), which is directly altered by the applied force.

## 3. Results and Discussion 

### 3.1. Effect of the Joint Angle on the Capacitive Sensor Output

The fingertip force sensors are attached to the distal areas of the glove, and measurements from the capacitive sensors can thus be influenced by either the longitudinal displacement of the sensor or finger tissue deformation, both of which are closely related to the finger posture. This section reports our experimental investigation of the joint angle effects (for the metacarpophalangeal (MCP)/proximal interphalangeal (PIP) joints of the index and middle fingers and the interphalangeal (IP)/adduction joints of the thumb) on the capacitive sensor output characteristics. The finger postures tested in these experiments are listed in Table 1. The flexion angle is the deviation from the fully extended posture, and the adduction angle is the deviation from the fully abducted posture.

**Figure 4:**
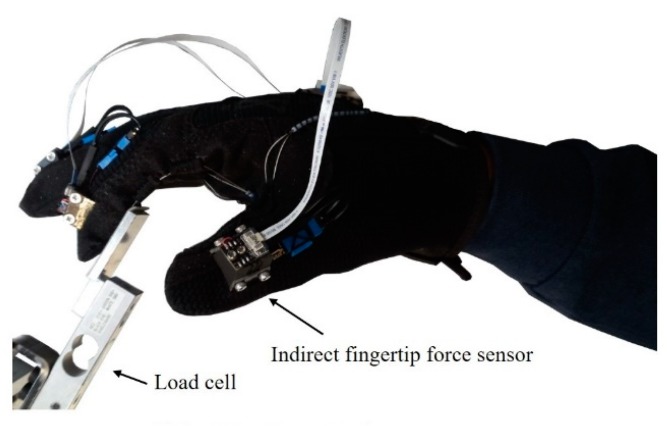
Experimental setup to measure fingertip force: the load cell measurement of the force exerted by the subject.

A load cell (651AL, KTOYO Co., Ltd., Uijeongbu, Korea) with a range of 0 to 10 kgf was attached to a fixture mounted on a table to measure the fingertip forces, as shown in Figure 4. The position and orientation of the fixture could be adjusted to align the force measurement plate that was attached to the load cell with the fingerpad of the wearer for each posture condition. The user exerted the force on the loadcell as if he pressed an object, as shown in Figure 2. In particular, the force measurement plate was adjusted to change the angle between the thumb and load cell when the user exerted a thumb shear force, as shown in Figure 2d. To exert a fingertip force during the thumb flexion and index/middle finger flexion, the user pressed the load cell, as shown in Figure 2c,e, and the user pressed the load cell, as shown in Figure 2d, to generate a thumb adduction force.

During the experiment, the user was requested to exert a fingertip force on the load cell by flexion/extension and abduction/adduction motions independently. The output signal from the load cell was measured at a sampling rate of 100 Hz. For each test condition, the following procedure was performed. The subject, who was seated in front of the table supporting the apparatus, placed a fingertip on the force measurement plate and adopted a specified posture with the aid of a protractor. The subject was instructed to exert a sinusoidal fingertip force that varied from 0 to 10 N with a frequency of 0.2 Hz on the force measurement plate. The sinusoidal signal was provided to the user through the monitor to provide visual information, and the subject applied the force according to the signal. The frequency of 0.2 Hz was chosen based on preliminary experiments on the capacitive sensor output characteristics and the effect of the loading and unloading frequency. There was no significant difference in the sensor output with frequencies of 0.05, 0.1, or 0.2 Hz, where 0.05 Hz was regarded as a quasi-static condition [37]. A frequency of 0.2 Hz was chosen based on the ease with which the subject was able to follow the cyclic sinusoidal loading and unloading cue. The force was exerted for three periods for each condition. The protocol (KH2016-62) was approved by the Institutional Review Board of Korea Advanced Institute of Science and Technology (KAIST). 

**Figure 5:**
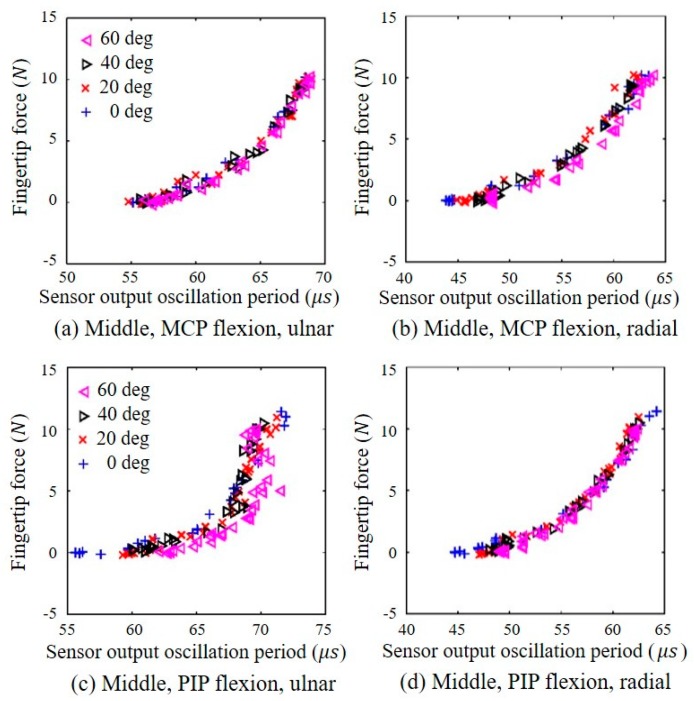
Relationship between the fingertip force (*N*) measured by the load cell and the fingertip force sensor output oscillation period (μs) measured by the proposed sensor for various joint angles (0, 20, 40, and 60 degrees) at the middle finger: relationship measured at (**a**) the ulnar side under MCP flexion, (**b**) the radial side under MCP flexion, (**c**) the ulnar side under PIP flexion, and (**d**) the radial side under PIP flexion.

Figure 5 shows the output oscillation periods of the output signal of the capacitive sensors versus the fingertip force for the various joint angles measured from the load cell. A longer sensor output oscillation period corresponds to a greater force applied to the sensor. As described by Equation (1), the capacitance becomes larger as the distance between the two plates decreases, and the sensor output oscillation period increases as the capacitance increases because the period is proportional to the capacitance, as described by Equation (2). In Figure 5, the results for the middle finger are shown because the data for the index and middle fingers are similar to the exponential function form. 

**Figure 6:**
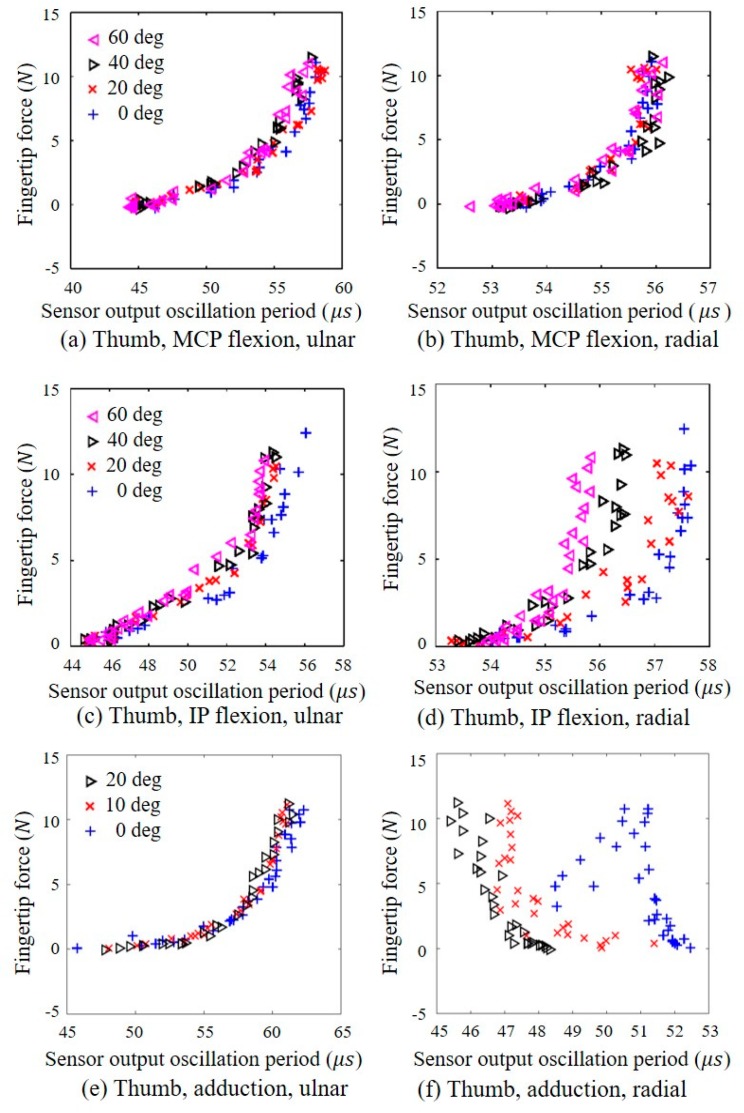
Relationship between the fingertip force (*N*) measured by the load cell and the fingertip force sensor output oscillation period (μs) measured by the proposed sensor for various joint angles (0, 20, 40, and 60 degrees for MCP and IP flexion and 0, 10, and 20 degrees for adduction) at the thumb: relationship measured at (**a**) the ulnar side under MCP flexion, (**b**) the radial side under MCP flexion, (**c**) the ulnar side under IP flexion, (**d**) the radial side under IP flexion, (**e**) the ulnar side under adduction, and (**f**) the radial side under adduction.

To identify the differences between the data for the middle and index fingers, a statistical analysis was performed using a repeated measures ANOVA test (*p* < 0.05). According to the statistical analysis, there were no significant differences between them (*p* > 0.05) regardless of the type of the fingers. The ulnar- and radial-side sensor outputs for MCP joint flexion of the middle finger are shown in Figure 5a,b, respectively. Figure 6a,b show the ulnar- and radial-side sensor outputs, respectively, for MCP joint flexion of the thumb. As shown in the graphs, the MCP joint angle does not have a significant effect on the sensor output characteristics. For flexion of the PIP joint of the middle finger, as presented in Figure 5c,d, neither the ulnar- nor the radial-side sensor exhibited a significant or consistent dependency on the PIP joint flexion angle when the fingertip force was less than approximately 5 N. In addition, the radial-side sensor exhibited less variation than the ulnar-side sensor for all PIP joint flexion angles. For IP joint flexion of the thumb, the output of the ulnar-side sensor was generally independent of the IP joint angle, as shown in Figure 6c, although the radial-side sensor output exhibited a notable joint angle dependency, as shown in Figure 6d. As shown in Figure 6e,f, the ulnar-side sensor output was generally independent of the thumb adduction angle. In contrast, the oscillation period of the radial-side sensor output decreased when either the fingertip adduction force or adduction angle increased, although the data were broadly distributed. This decrease in the output oscillation period indicated that the exertion of an adduction force or an increase in the adduction angle reduced the force measured by the radial-side sensor. Based on these observations, the outputs of the radial-side sensors on the index and middle fingers and the ulnar-side sensor on the thumb were selected to estimate the flexion force to ensure that the PIP and IP joint angles had a minimal effect. To estimate the thumb adduction force, the ulnar-side sensor on the thumb was selected because the output of this sensor was generally independent of the adduction force. In the case of the thumb, the direction of the force can be identified by comparing the signs of the changes in the oscillation periods of the ulnar- and radial-side sensor outputs. Figure 6a,c,e indicates that the ulnar-side sensor output oscillation period increases as either the flexion or adduction force increases. In contrast, the output oscillation period of the thumb radial-side sensor increases with increasing force during flexion (Figure 6b,d,f) but decreases with an increase in the adduction force.

### 3.2. Calibration to the Fingertip Force

**Figure 7:**
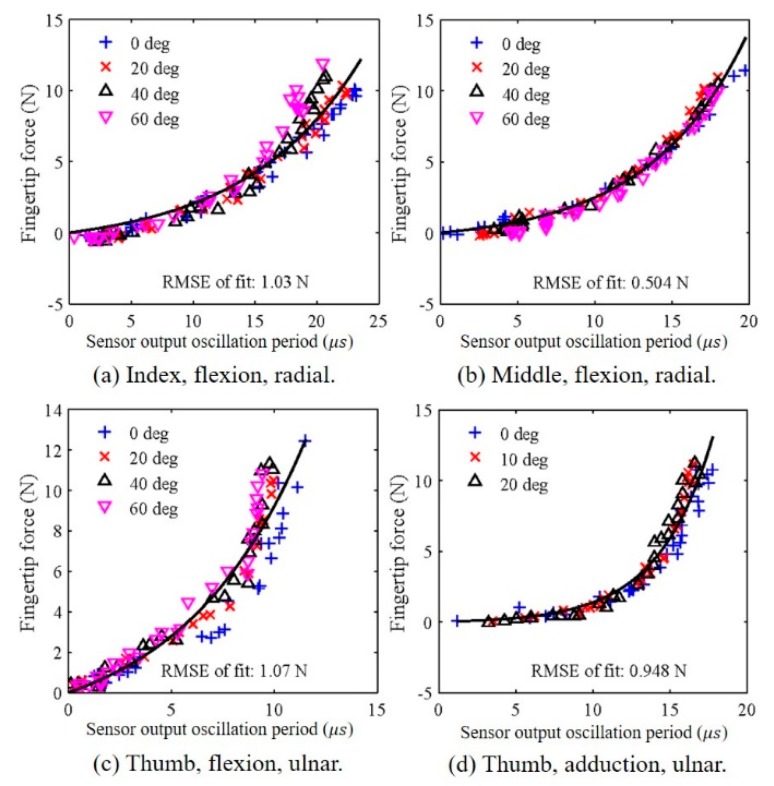
Calibration results of the relationship between the fingertip force measured by the load cell and the fingertip force sensor output oscillation period with offset removed for different levels of flexion force of (**a**) the index finger and (**b**) the middle finger. Sensor output oscillation periods for different levels of (**c**) flexion force of the thumb and (**d**) adduction force of the thumb.

The selected sensors (the radial-side sensors on the index and middle fingers and the ulnar-side sensor on the thumb were calibrated to the fingertip force based on the fingertip force measured data (Figure 7). The effects of posture were disregarded; thus, the datasets for the various joint angle conditions were combined for each DoF. The sensor output oscillation periods were processed using an offset removal algorithm, where the offset for each sensor output was the shortest period measured during the exertion of the flexion force for PIP or IP joint angles of 0°, 20°, 40°, and 60°. The sensor offsets for thumb adduction were similar to those for thumb flexion. Based on the results of force estimation using the relationships between the force and the sensor output oscillation period, the following expression was selected to calibrate the sensors:

(3) $$y=a(e^{bx}-1),$$

where *y* and *x* are the fingertip force (flexion or adduction) and the sensor output with the offset removed, respectively, and *a* and *b* are constants that must be individually obtained via calibration for each wearer. This functional form was chosen such that the fingertip force, *y*, was 0 for a sensor output offset, *x*, of 0.

The functions obtained based on Equation (3) are presented in Figure 7 as solid curves. The goodness of fit, expressed as the root mean square error (RMSE), is presented for each curve. The resulting parameters for each finger motion are presented in Table 2. As shown in Figure 7a, the RMSE of the index finger flexion force was low when the force was relatively weak (the RMSE was 0.843 N below approximately 5 N), regardless of the finger posture. However, for larger forces, the error tendencies differed for different finger postures. For large flexion angles, the RMSE was high, and the error increased as the angle increased. Moreover, the estimation accuracies obtained below 5 N in this study showed better performance compared with our previous study using load cells (0.653 N, RMSE for the index finger) [45] and other study (1.179 N, mean error for the index finger) [41]. For the middle finger (Figure 7b), the errors were low for all flexion angles and periods. In particular, the RMSE was 0.261 N below approximately 5 N. For thumb flexion (Figure 7c), the RMSE tended to be low when the thumb was fully extended and when the fingertip force was larger than approximately 2.5 N (the RMSE was 0.796 N below approximately 5 N). When the thumb flexion force was greater than approximately 8 N, the RMSE tended to be high, except for the fully extended posture. For thumb adduction (Figure 7d), the goodness of fit tended to be somewhat high regardless of the finger posture when the fingertip force was relatively small (the RMSE was 0.387 N below approximately 5 N). For greater adduction forces, the RMSE tended to be high, except for the fully abducted posture in which case the goodness of fit was high.

Although the calibration process is simple, two minor factors must be considered. First, the fingertip force sensor must be recalibrated after removing the glove and putting it back on again. During recalibration, adjustability problems occur because the glove is not rigid. The finger does not fit perfectly to the sensor, and the previous alignment between the finger and the sensor cannot be exactly matched when putting the system on again. It is difficult to position the finger exactly in the middle of the capacitive sensors. However, the current calibration process is not complicated; thus, it is easy to recalibrate and solve this adjustability issue. During calibration, constants *a* and *b* indicated in Equation (3) must be calculated. As shown in Figure 7, the relationship between the sensor output oscillation period and the fingertip force measured at the capacitive sensor does not vary with the joint angles; therefore, *a* and *b* can be calculated from one instance of the user holding an object.

Second, the calibration is performed for only one DoF at a time for thumb adduction/abduction and flexion/extension. The fingertip force can only be measured when performing these two actions independently because only the sensor value on the ulnar side varies during adduction/abduction, whereas the sensor values for the ulnar and radial sides vary simultaneously during flexion/extension, as shown in Figure 6c,d. According to previous research, the total grasp taxonomy consists of 33 grasp actions. Only seven of them (lateral tripod, tripod variation, tripod, quadpod, precision disk, precision sphere, and writing tripod) require motion in two DoFs at the same time [51]. For these seven actions, the fingertip force can be measured if the user grasps the object consciously such that the motion does not occur in both DoFs simultaneously. Therefore, the user can perform approximately 80% or more of all grasping actions using this developed system.

### 3.3. Accuracy of the Fingertip Force Measurement

**Figure 8:**
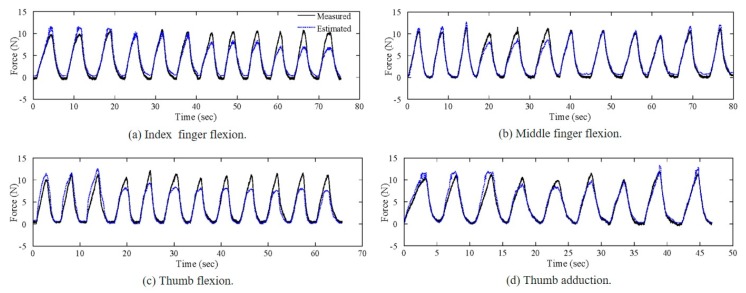
Measured and calibrated fingertip forces for (**a**) index finger flexion, (**b**) middle finger flexion, (**c**) thumb flexion, and (**d**) thumb adduction.

Calibration accuracy is discussed in this section. The parameters and conditions were identical to those in the experiments regarding the effects of the PIP and IP joint flexion angles and the thumb adduction angle on the sensor output characteristics. Data were processed to remove the offsets, and the estimated fingertip force was calculated based on Equation (3) using the values for parameters *a* and *b* given in Table 2. The calibrated fingertip force was compared to the measured fingertip force from the load cell to calculate the calibration error in terms of the RMSE. Figure 8 presents the measured and calibrated forces, where the solid and dashed lines represent the measured and calibrated values, respectively. Each subfigure presents the merged data for all of the posture conditions for each specific finger. For example, the data for the first three periods among the twelve shown in Figure 8a correspond to a PIP joint angle of 0°, and the next three periods correspond to a 20° angle. Table 3 presents the RMSE values for the calibrated fingertip forces for each posture condition and each finger in units of N. The calibration accuracy varied with the posture. Although the effect of finger posture was disregarded in the fingertip force measurements, the finger posture affected the sensor outputs and calibration accuracy. For index finger flexion (Figure 8a), the calibration accuracy was high when the force was smaller than approximately 5 N, regardless of the finger posture. At forces greater than 5 N, however, the calibration error depended on the posture; specifically, the error increased as the finger flexion angle increased. The highest overall calibration accuracy was obtained for middle finger flexion (Figure 8b), although the accuracy was low when the flexion angle was 20°. In the case of thumb flexion (Figure 8a), the accuracy was high when the thumb was fully extended, whereas the accuracy was low at other flexion angles. Overall, the calibration accuracy was lowest for thumb flexion, whereas for thumb adduction (Figure 8d), the accuracy was high for the fully abducted posture and low for the 10° adducted posture. The errors were generally as expected, except that the calibration accuracy for the middle finger was unexpectedly low at a flexion angle of 20°. There were minor errors in the force measurements because the finger posture affected the sensor outputs, and the error may also depend on the specific user. The size and the mechanical properties (e.g., skin stiffness) of the fingertip can affect the sensor performance. For example, the offset of the sensor output oscillation period depends on the degree how much the fingertip fits to the sensor. Moreover, the relationship between the fingertip force and the expansion of the tissue is important for the performance of our proposed sensors. Considering those human factors (size and mechanical properties of the fingertip), we are going to introduce a more complex force calibration method that includes finger posture as an input variable to reduce the estimation errors. Moreover, the side-mounted planar force sensors could be replaced with thin force sensors embedded around the perimeter of the open-pad structure. This modification should minimize the dependency of the fingertip force measurement performance on the finger posture.

## 4. Conclusions

In this paper, we presented a fingertip force sensor that is intended for use with glove-type assistive devices. The fingertip sensor were designed to measure the fingertip force for flexion of the index and middle fingers and flexion and adduction of the thumb. Openings on the palmar surface of each finger allowed direct contact between the fingerpads of the wearer and the object being manipulated. Capacitive sensors were mounted on the sides of the fingers and the thumb to sense force, and a multi-vibrator circuit based on a Schmitt trigger inverter was used to measure the change in the capacitance based on the oscillation frequency. These sensors demonstrated the ability to measure the grip force for various posture conditions. The accuracy of the fingertip force measurements ranged from 3.75 to 9.71% after calibration, regardless of the finger joint angles or the finger posture.

In the future, we plan to introduce a more complex force estimation method to reduce the estimation error and increase the robustness to disturbances by improving the structure of the sensor. For example, a support that fills the space between the sensor and the finger will be designed by a 3D printer so that the position of the sensor is not changed or warped when the user wears the sensor. In addition, to integrate the proposed sensor with a glove-type assistive device, the design of the side-mounted planar force sensors could be improved with thin force sensors embedded in the perimeters of the fingerpad openings depending on the glove design. We will validate the performance of individual actuated finger movements driven by an assistive device using the proposed sensor.

## Figures and Tables

**Table 1:** Experimental finger posture conditions in units of degrees (°).

Posture	MCP Flexion	PIP/IP Flexion	Adduction
Index, MCP flexion	0, 20, 40, 60	15	0
Middle, MCP flexion	0, 20, 40, 60	15	0
Thumb, MCP flexion	30, 50, 70, 90	10	0
Index, PIP flexion	15	0, 20, 40, 60	0
Middle, PIP flexion	15	0, 20, 40, 60	0
Thumb, IP flexion	10	0, 20, 40, 60	0
Thumb, adduction	90	10	0, 10, 20

**Table 2:** Parameters for fingertip force estimation and goodness of fit (RMSE, in N).

Finger, Direction	a	b	Goodness of Fit (RMSE, in N)
Index, flexion	1.055	0.1074	1.0335
Middle, flexion	0.6605	0.156	0.5043
Thumb, flexion	2.129	0.1673	1.0695
Thumb, adduction	0.08567	0.2835	0.9478

**Table 3:** Fingertip force estimation performance.

Finger, Direction	Joint Angle (°)	Estimation Error (RMSE, in N)
Index, flexion	PIP: 0	0.876
	PIP: 20	0.689
	PIP: 40	1.123
	PIP: 60	1.639
	Overall	1.123
Middle, flexion	PIP: 0	0.507
	PIP: 20	1.093
	PIP: 40	0.449
	PIP: 60	0.615
	Overall	0.716
Thumb, flexion	IP: 0	1.571
	IP: 20	1.030
	IP: 40	1.002
	IP: 60	1.453
	Overall	1.290
Thumb, adduction	0	1.209
	10	0.703
	20	0.552
	Overall	0.868
